# Analysing diet of small herbivores: the efficiency of DNA barcoding coupled with high-throughput pyrosequencing for deciphering the composition of complex plant mixtures

**DOI:** 10.1186/1742-9994-6-16

**Published:** 2009-08-20

**Authors:** Eeva M Soininen, Alice Valentini, Eric Coissac, Christian Miquel, Ludovic Gielly, Christian Brochmann, Anne K Brysting, Jørn H Sønstebø, Rolf A Ims, Nigel G Yoccoz, Pierre Taberlet

**Affiliations:** 1 Department of Biology, University of Tromsø, N-9037 Tromsø, Norway; 2 Laboratoire d'Ecologie Alpine, CNRS-UMR 5553, Université Joseph Fourier, BP 53, 38041 Grenoble Cedex 09, France; 3 National Centre for Biosystematics, Natural History Museum, University of Oslo, PO Box 1172 Blindern, N-0318 Oslo, Norway; 4 Centre for Ecological and Evolutionary Synthesis, Department of Biology, University of Oslo, PO Box 1066 Blindern, N-0316 Oslo, Norway

## Abstract

**Background:**

In order to understand the role of herbivores in trophic webs, it is essential to know what they feed on. Diet analysis is, however, a challenge in many small herbivores with a secretive life style. In this paper, we compare novel (high-throughput pyrosequencing) DNA barcoding technology for plant mixture with traditional microhistological method. We analysed stomach contents of two ecologically important subarctic vole species, *Microtus oeconomus *and *Myodes rufocanus*, with the two methods. DNA barcoding was conducted using the P6-loop of the chloroplast *trn*L (UAA) intron.

**Results:**

Although the identified plant taxa in the diets matched relatively well between the two methods, DNA barcoding gave by far taxonomically more detailed results. Quantitative comparison of results was difficult, mainly due to low taxonomic resolution of the microhistological method, which also in part explained discrepancies between the methods. Other discrepancies were likely due to biases mostly in the microhistological analysis.

**Conclusion:**

We conclude that DNA barcoding opens up for new possibilities in the study of plant-herbivore interactions, giving a detailed and relatively unbiased picture of food utilization of herbivores.

## Background

Small mammalian herbivores, such as voles and lemmings, play a key role in many boreal ecosystems where they function as the main link between the vegetation and predators [1,2]. Their dramatic population fluctuations and consequent ecosystem implications [3,4] have been subject to intensive studies for decades [5-7]. Trophic interactions have been emphasized as a main determinant of these fluctuations [8]. While predator-prey interactions have been much emphasized [6], the interactions between voles and plants have gained new attention lately (see e.g. [9,10]), as new interaction pathways have been identified [11-13]. However, plant-herbivore interactions cannot be understood without knowing, among other aspects, herbivore diets in natural settings. Yet most studies on plant-vole interactions have used only limited and indirect diet data [14-16], such as recording signs of rodent feeding on vegetation [17,18] or cafeteria experiments in artificial settings [19,20]. The most direct information comes from microhistological analysis of stomach content [21-24]. However, this is a very time-consuming method giving unspecific and context-dependent results [25], due to the small particle size as well as the composition of encountered plant material in rodent stomachs.

DNA barcoding, i.e. taxon identification using a standardized DNA region [26], is now increasingly used in ecological studies (see Valentini et al. [27] for a review), including diet analysis. DNA barcoding is particularly useful in diet determination when the food is not identifiable by morphological criteria, such as in the case of liquid feeders [28], or when the diet cannot be deduced by observing the feeding behavior, (e.g. diatom-feeding krill [29]). Diet analyses based on DNA markers concern so far mostly carnivorous animals (e.g. [30]). A universal approach for herbivorous diet analysis has been developed recently [31]. This approach combines the new highly parallel sequencing systems [32], with the amplification of the P6 loop of the chloroplast *trn*L (UAA) intron [33].

In this paper, we present the first results on species-level food selection of two ecologically important vole species (*Microtus oeconomus *and *Myodes *(formerly *Clethrionomys*) *rufocanus*) in sub-arctic ecosystems based on DNA barcoding of ingested plants. We compare the results with the traditional microhistological method, which, for small rodents, has previously not been assessed against other methods.

## Methods

### Vole trapping

The voles were sampled in low arctic tundra at Varanger peninsula (Finnmark, Norway: 70° 20' N, 30° 00' E) in July and September 2007. The sampling was conducted by snap trapping according to standard methods [34], after which the voles were dissected and their stomachs were stored in 70% ethanol for approximately 1/2 year prior to analysis. A sample of 48 individuals was chosen to be analyzed with both methods, based on the available space in DNA analysis batch and on stratifying according to species and season. The stomachs were dissected and the contents homogenized. Two subsamples were taken from each stomach for DNA analysis. They were stored in paper filter bags and submerged in silicagel to dry them. Remaining stomach contents were stored in 70% ethanol until the microhistological analysis.

### Microhistological analysis

Microhistological recognition of food particles in vole stomachs was based on leaf epiderm morphology. The shape of epidermal cells is taxon-specific, and several additional features, such as trichomes, hairs and characteristics of cells surrounding stomata, can be used for species identification [25,35]. A photography-guide (Soininen & Nielsen, unpublished data) of epidermis of all vascular plants recorded at the sampling area (Ravolainen *et al*. unpublished data) was prepared using a method modified from Carrière [25]. Dry plant samples were soaked overnight, scraped to reveal the epidermis and bleached with household bleach to clear the tissue of chlorophyll. Hard leaves were first boiled in table vinegar to soften the mesophyll tissue. Microphotographs (40×) were taken of abaxial and adaxial leaf side and leaf edge of all plants. Additional photographs were taken of stems and seeds of certain species of special interest. In addition to the specifically prepared epidermis photographs, photographs and microscopy slides of arctic plant epidermis, received from C. Hübner and E. Bjørkevoll, were used to aid identification.

A method modified from Hansson [36] was used for microscopy analysis. After taking subsamples for DNA analysis, stomach contents were filtered to > 0.16 mm and > 0.56 mm fractions. These were bleached with approximately 2 mL of household bleach for approximately 1/2 hour. One sample per fraction was analyzed, mounting a droplet of it on a microscopy slide. The frequency of occurrence of food items was recorded by light microscope (40×), by counting 25 hits on identifiable material along a measure grid. When approximately 95% of fragments were unidentifiable, the slide was discarded. For four individuals, no slide with adequate amount of identifiable material could be made. Therefore, they were discarded from the microhistological analysis.

### DNA analysis

Two samples were analyzed for each individual. Total DNA was extracted from about 10 mg of sample with the DNeasy Tissue Kit (Qiagen GmbH, Hilden, Germany), following the manufacturer's instructions. The DNA extracts were recovered in a total volume of 300 *μ*L. Mock extractions without samples were systematically performed to monitor possible contaminations.

DNA amplifications were carried out in a final volume of 25 *μ*L, using 2.5 *μ*L of DNA extract as template. The amplification mixture contained 1 U of AmpliTaq^® ^Gold DNA Polymerase (Applied Biosystems, Foster City, CA), 10 mM Tris-HCl, 50 mM KCl, 2 mM of MgCl_2_, 0.2 mM of each dNTP, 0.1 *μ*M of each primer, and 0.005 mg of bovine serum albumin (BSA, Roche Diagnostic, Basel, Switzerland). The mixture was denatured at 95°C for 10 min, followed by 35 cycles of 30 s at 95°C, and 30 s at 55°C; the elongation was removed in order to reduce the +A artifact [37,38]. Samples were amplified with using the universal primers *g *and *h *described by Taberlet et al. [33]. The addition of a specific tag on the 5' end allowed an assignement of sequences to the respective samples. After amplification all samples were pooled for the pyrosequencing run. Each sample was recognized by a specific five bases long tag with at least two differences between tags for a better assignation of sequences to samples during bioinformatic segregation of sequences.

PCR products were purified using the MinElute PCR purification kit (Qiagen GmbH, Hilden, Germany). DNA quantification was carried out using the BioAnalyzer (Agilent Technologies, Inc., Santa Clara, CA). Taking these concentrations into account PCR products were pooled leading to equal amounts per sample. Large-scale pyrosequencing was carried out using GS FLX sequencer (Roche, Basel, Switzerland) following the manufacturer's instructions.

The first step of analyzing the output of the pyrosequencing consisted of sorting the different sequences according to the tag present on the 5' end of the primers. Thus, for each sample (each stomach content), a new file was generated, containing all the sequences having the relevant tag. Then, these sequences were analyzed to determine the diet. To limit the influence of sequence errors [39], only sequences that were present more than three times were considered in the subsequent analyses.

The sequences were compared to a database of 842 species representing all widespread and/or ecologically important taxa of the arctic flora (GenBank accession number GQ244527 to GQ245667) (Sønstebø et al: A minimalist DNA barcoding approach for reconstructing past Arctic vegetation and climate, submitted). It was developed by sequencing the whole chloroplast *trn*L (UAA) intron of these species using primer pair designed by Taberlet et al. [40], and following the protocol described and evaluated in Taberlet et al. [33]. In the database a total of 33,5% of species and 77,1% of genera could be identified by the P6 loop. All families were unambiguously identified (Sønstebø et al: A minimalist DNA barcoding approach for reconstructing past Arctic vegetation and climate, submitted). When sequences were not fully identified using the arctic plant database, they were compared with sequences retrieved from GenBank, using ecoPCR [33]; . The taxon was assigned to each sequence in a dataset by similarity assessment with a reference database using FASTA [41] algorithm. A FASTA alignment was retrieved if there was at least 98% of identity between query and database sequences and 100% of query coverage. If two or more taxa could be assigned with the same score for a given sequence, we assigned this sequence to the higher taxonomic level that included both taxa. This method resulted in some sequenced taxa being assigned to the rank of genus or family.

Chimeric sequences are a well know problem when amplifying a mixture of homologous genes, and it is impossible to avoid their formation [42]. But if two unrelated taxa compose the chimeric sequence the resulting sequence is not taken into account because for taxon identification the FASTA alignments is retrieved only if the sequence have 100% of query coverage with the reference sequence. If two related taxa compose the chimeric sequence, this sequence is assigned to the higher taxonomic level that included both taxa (e.g. genus, family, order, etc.).

### Data analysis

Results of the two methods were compared in two ways. First, the taxonomic resolution obtained by the two methods was examined by comparing the relative frequencies of hits (microscopy) and sequences (DNA) at different taxonomic levels. For this comparison, the four individuals with no microscopy data were excluded also from DNA dataset.

Second, the relative frequencies of food items in diets were compared between the methods. Taxonomic adjustments were first made to make the results from the two methods comparable. Nomenclature from species to family level follows Lid & Lid [43] and Elven [44], and for higher taxonomy Judd et al. [45]. Several genera are represented only by one species in the study area and sequences assigned to these were therefore attributed to the respective species (e.g. *Arctous alpinus*, *Bistorta vivipara*, *Chamaepericlymenum suecicum*, *Rumex acetosa*). Similar adjustments were done at other taxonomic levels (e.g. Salicaceae to *Salix *and Ranunculales to Ranunculaceae). Then, proportions of food items estimated at the level of individual voles were averaged (mean ± standard deviation) across species and sampling season for both methods.

## Results

**Figure 1:**
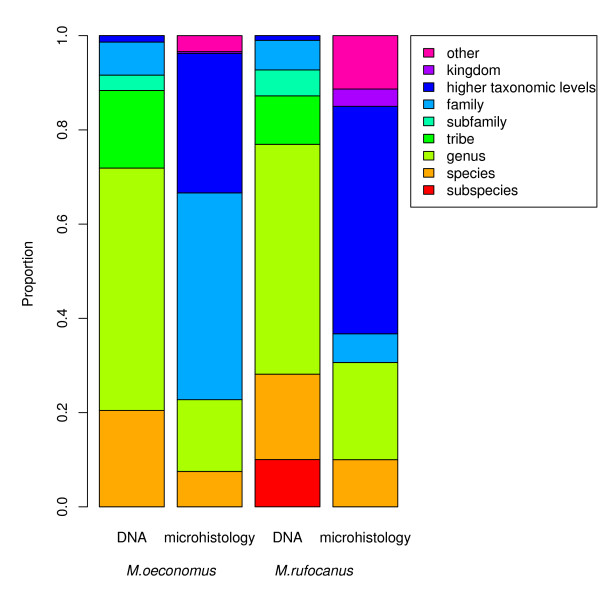
**Taxonomic resolution of vole diets**. Taxonomic resolution of the diets of *Microtus oeconomus *and *Myodes rufocanus *according to two methods of diet analysis (microhistology and DNA barcoding). Proportions are taken from total number of hits on identifiable material for microhistology and total number of identified sequences for DNA barcoding. Category "other" includes items not assigned to any taxonomic level by microhistology, but to morphologic groups (e.g. seed, root).

Using DNA barcoding, 75% of all sequences were identified at least to the genus level (Figure 1), whereas with the microhistological method, less than 20% of the identified fragments could be specified at this level. Consequently, more plant species and genera were identified in vole diets with the DNA barcoding than microhistology (Table 1 and 2). For the *M. oeconomus *diet, DNA barcoding identified 13 species and 9 genera compared with 9 and 5 with microscopy. Corresponding numbers for *M. rufocanus *were 17 and 8 (DNA barcoding) compared with 11 and 7 (microscopy).

**Table 1:** Diet of Microtus oeconomus

season method	summer dna (N = 14)	mic (N = 13)	autumn dna (N = 4)	mic (N = 4)
*Anthoxanthum nipponicum*	.01 (± .02)	.005 (± .02)	.03 (± .03)	.005 (± .01)
*Avenella flexuosa*	.01 (± .01)	.01 (± .04)	.04 (± .06)	.02 (± .03)
*Bistorta vivipara*	.03 (± .10)	.005 (± .02)	.06 (± .08)	0
*Chamaepericlymenum suecica*	0	0	.01 (± .01)	0
*Cirsium heterophyllum*	.01 (± .03)	.01 (± .02)	0	0
*Deschampsia cespitosa*	.02 (± .03)	.02 (± .04)	0	.01 (± .01)
*Oxyria digyna*	0	.01 (± .02)	0	0
*Phleum alpinum*	.01 (± .02)	0	.04 (± .04)	0
*Poa alpina*	0	NA	.005 (± .01)	NA
*Rumex acetosa*	.37 (± .25)	.002 (± .01)	.18 (± .18)	0
*Salix herbaceae*	0	.001 (± .01)	0	.005 (± .01)
*Solidago virgaurea*	0	.002 (± .01)	0	.005 (± .01)
*Stellaria nemorum*	.003 (± .01)	NA	0	NA
*Trientalis europaea*	.07 (± .23)	NA	.07 (± .15)	NA
*Trollius europeus*	0	NA	.02 (± .04)	NA
*Viola biflora*	.02 (± .04)	NA	.01 (± .02)	NA

genus				

*Anthoxanthum*	.01 (± .02)	.005 (± .02)	.03 (± .03)	.005 (± .01)
*Avenella*	.01 (± .01)	.01 (± .04)	.04 (± .06)	.02 (± .03)
*Bistorta*	.03 (± .10)	.005 (± .02)	.06 (± .08)	0
*Calamagrostis*	.05 (± .14)	NA	.02 (± .02)	NA
*Cerastium*	.04 (± .08)	NA	0	NA
*Chamaepericlymenum*	0	0	.01 (± .01)	0
*Cirsium*	.01 (± .03)	.01 (± .02)	0	0
*Deschampsia*	.02 (± .03)	.02 (± .04)	0	.01 (± .01)
*Festuca*	.01 (± .02)	.03 (± .07)	.03 (± .04)	.01 (± .01)
*Geranium*	0	NA	.01 (± .03)	NA
*Oxyria*	0	.01 (± .02)	0	0
*Poa*	0	.01 (± .01)	.005 (± .01)	0
*Phleum*	.01 (± .02)	0	.04 (± .04)	0
*Ranunculus*	.10 (± .12)	NA	.08 (± .06)	NA
*Rumex*	.37 (± .25)	.002 (± .01)	.18 (± .18)	0
*Salix*	.16 (± .15)	.01 (± .03)	.08 (± .12)	.02 (± .03)
*Solidago*	0	.002 (± .01)	0	.005 (± .01)
*Stellaria*	.003 (± .01)	NA	0	NA
*Trientalis*	.07 (± .23)	NA	.07 (± .15)	NA
*Trollius*	0	NA	.02 (± .04)	NA
*Vaccinium*	0	0	.10 (± .13)	.005 (± .01)
*Viola*	.03 (± .06)	0	.01 (± .02)	0
*Equisetum*	.01 (± .02)	.15 (± .23)	.01 (± .02)	.01 (± .01)

family				

Apiaceae	.01 (± .04)	NA	0	NA
Asteraceae	.02 (± .04)	.01 (± .03)	.07 (± .13)	.01 (± .01)
Caryophyllaceae	.05 (± .10)	.05 (± .12)	0	.06 (± .08)
Cornaceae	0	0	.01 (± .01)	0
Cyperaceae	0	.01 (± .03)	0	.02 (± .04)
Geraniaceae	0	NA	.01 (± .03)	NA
Ericaceae	0	0	.12 (± .16)	.01 (± .01)
Poaceae	.11 (± .13)	.33 (± .35)	.21 (± .20)	.31 (± .23)
Polygonaceae	.43 (± .29)	.16 (± .17)	.26 (± .13)	.04 (± .08)
Primulaceae	.07 (± .23)	NA	.07 (± .15)	NA
Ranunculaceae	.11 (± .13)	NA	.10 (± .10)	NA
Rosaceae	0	.002 (± .01)	.03 (± .06)	0
Salicaceae	.16 (± .15)	.01 (± .03)	.08 (± .12)	.02 (± .03)
Scrophulariaceae	.001 (± .01)	0	0	0
Violaceae	.03 (± .06)	0	.01 (± .02)	0
Equisetaceae	.01 (± .02)	.15 (± .23)	.01 (± .02)	.01 (± .01)

higher taxonomic levels				

Eudicots	.88 (± .14)	.47 (± .37)	.78 (± .19)	.54 (± .23)
Monocots	.11 (± .13)	.35 (± .36)	.21 (± .19)	.35 (± .29)
Monilophytes	.01 (± .02)	.15 (± .23)	.01 (± .02)	.01 (± .01)
Euphyllophytes	0	.02 (± .03)	0	.09 (± .13)
Bryophytes	NA	.01 (± .02)	NA	.01 (± .02)
Fungi	NA	0	NA	.01 (± .03)

Proportion of identified food items in the diet of *Microtus oeconomus *using microhistological (mic) and DNA barcoding (dna) methods, summer and autumn, (mean ± standard deviation). At each taxonomic level, also the proportions from lower levels are included, except for Euphyllophytes where only the items which could not be assigned to a lower taxa are included. NA = Food item not identifiable with the method in question.

**Table 2:** Diet of Myodes rufocanus

season method	summer dna (N = 18)	Mic (N = 16)	autumn dna (N = 12)	mic (N = 11)
*Arctous alpinus*	.002 (± .01)	NA	0	NA
*Avenella flexuosa*	.01 (± .02)	.01 (± .03)	0	0
*Bistorta vivipara*	.05 (± .11)	0	.01 (± .05)	0
*Chamaepericlymenum suecica*	.09 (± .17)	.004 (± .01)	.003 (± .01)	0
*Deschampsia cespitosa*	.01 (± .06)	0	0	.01 (± .02)
*Empetrum nigrum hermafroditum*	.12 (± .18)	.01 (± .03)	.05 (± .07)	0
*Oxycoccos microcarpus*	0	NA	.001 (± .004)	NA
*Oxyria digyna*	0	.003 (± .01)	0	0
*Pedicularis lapponica*	0	.004 (± .01)	0	0
*Phleum alpinum*	.04 (± .04)	0	.09 (± .06)	0
*Pyrola minor*	.0003 (± .001)	NA	0	NA
*Rumex acetosa*	.08 (± .12)	.004 (± .013)	.05(± .08)	0
*Salix herbaceae*	0	.01 (± .03)	0	0
*Solidago virgaurea*	.01 (± .04)	0	0	0
*Trientalis europaea*	.01 (± .02)	NA	.003 (± .01)	NA
*Trollius europaeus*	.002 (± .01)	NA	0	NA
*Vaccinium myrtillus*	.001 (± .003)	0	.01 (± .01)	.04 (± .05)
*Vaccinium uliginosum microphyllum*	.05 (± .06)	.02 (± .02)	.19 (± .31)	.01 (± .03)
*Vaccinium vitis-idaea*	.01 (± .03)	.01 (± .02)	0	.00 (± .01)
*Viola biflora*	.01 (± .05)	NA	0	NA

genus				

*Arctous*	.002 (± .01)	NA	0	NA
*Avenella*	.01 (± .02)	.01 (± .03)	0	0
*Betula*	.01 (± .02)	NA	.04 (± .14)	NA
*Bistorta*	.05 (± .11)	0	.01 (± .05)	0
*Chamaepericlymenum*	.09 (± .17)	.004 (± .01)	.003 (± .01)	0
*Deschampsia*	.01 (± .06)	0	0	.01 (± .02)
*Empetrum*	.12 (± .18)	.01 (± .03)	.05 (± .07)	0
*Epilobium*	.01 (± .03)	NA	0	NA
*Festuca*	0	.01 (± .02)	0	0
*Geranium*	.002 (± .01)	NA	.04 (± .12)	NA
*Hierachium*	0	0	0	.004 (± .01)
*Oxycoccos*	0	NA	.001 (± .004)	NA
*Oxyria*	0	.003 (± .01)	0	0
*Pedicularis*	0	.004 (± .01)	0	0
*Phleum*	.04 (± .04)	0	.09 (± .06)	0
*Pyrola*	.0003 (± .001)	NA	0	NA
*Ranunculus*	.01 (± .05)	NA	.02 (± .08)	NA
*Rumex*	.08 (± .12)	.004 (± .013)	.05(± .08)	0
*Salix*	.13 (± .24)	.03 (± .05)	.02 (± .04)	.01 (± .04)
*Solidago*	0	.01 (± .04)	0	0
*Trientalis*	.01 (± .02)	NA	.003 (± .01)	NA
*Trollius*	.002 (± .01)	NA	0	NA
*Vaccinium*	.22 (± .16)	.19 (± .13)	.49 (± .33)	.19 (± .16)
*Viola*	.01 (± .05)	.002 (± .01)	0	0
*Equisetum*	.06 (± .22)	.11 (± .24)	.005 (± .02)	.02 (± .05)
*Gymnocarpium*	.002 (± .01)	NA	0	NA

family				

Apiaceae	0	NA	.003 (± .01)	NA
Asteraceae	.03 (± .09)	.01 (± .04)	.03 (± .07)	.004 (± .01)
Betulaceae	.01 (± .02)	NA	.04 (± .14)	NA
Caryophyllaceae	0	.001 (± .01)	0	.03 (± .05)
Cornaceae	.09 (± .17)	.004 (± .01)	.003 (± .01)	0
Cyperaceae	.004 (± .01)	.001 (± .01)	0	.001 (± .01)
Ericaceae	.42 (± .34)	.20 (± .13)	.61 (± .32)	.19 (± .16)
Geraniaceae	.002 (± .008)	.001 (± .005)	.04 (± .12)	.03 (± .05)
Onagraceae	.01 (± .03)	NA	0	NA
Orobanchaceae	0	.004 (± .01)	0	0
Poaceae	.07 (± .06)	.03 (± .08)	.14 (± .13)	.04 (± .12)
Polygonaceae	.14 (± .20)	.03 (± .04)	.07 (± .09)	.01 (± .02)
Primulaceae	.01 (± .02)	NA	.003 (± .01)	NA
Pyrolaceae	.0003 (± .001)	NA	0	NA
Ranunculaceae	.01 (± .06)	NA	.03 (± .09)	NA
Salicaceae	.13 (± .24)	.03 (± .05)	.02 (± .04)	.01 (± .04)
Scrophulariaceae	0	.01 (± .02)	0	0
Violaceae	.01 (± .05)	.003 (± .01)	0	0
Equisetaceae	.06 (± .22)	.11 (± .24)	.005 (± .02)	.02 (± .05)
Polypodiaceae	.002 (± .01)	NA	0	NA

higher taxonomic levels				

Eudicots	.86 (± .23)	.80 (± .28)	.86 (± .12)	.63 (± .15)
Monocots	.07 (± .07)	.04 (± .11)	.14 (± .13)	.04 (± .12)
Monilophytes	.06 (± .22)	.11 (± .24)	.005 (± .02)	.02 (± .05)
Euphyllophytes	.004 (± .02)	.03 (± .05)	0	.22 (± .11)
Bryophytes	NA	.01 (± .04)	NA	.01 (± .01)
Fungi	NA	.003 (± .01)	NA	.09 (± .13)

Proportion of identified food items in the diet of *Myodes rufocanus *using microhistological (mic) and DNA-barcoding (dna) methods, summer and autumn, (mean ± standard deviation). At each taxonomic level, also the proportions from lower levels are included, except for Euphyllophytes where only the items which could not be assigned to a lower taxa are included. NA = Food item not identifiable with the method in question.

Differences in taxonomic resolution between the methods made diet comparison complicated. For instance using microhistology, a large number of fragments could only be assigned to high taxonomic levels such as eudicotyledons and monocotyledons. Still, most of the species or genera that according to DNA barcoding were found to be quantitatively important in the diet, were also found to be so in the microhistological data, after differences in taxonomic accuracy were taken into account (Table 1 and 2). For example, for *M. rufocanus *both methods suggested that *Vaccinium *spp. are important food resources during both seasons, but especially in autumn (Table 2). Similarly, there was an agreement between the methods with respect to the importance of the family Polygonaceae (including *Rumex acetosa *and other species within this family) for both vole species in summer and that family Caryophyllaceae (with the species *Stellaria nemorum *and *Cerastium *spp.) is prevalent in *M. oeconomus *diet in the same season (Table 1 and 2). On the contrary, the substantial amounts of graminoids and horsetails (*Equisetum *spp.) found in the diet of *M. oeconomus *with microscopy were not evident from DNA analysis. Moreover, the prevalence of non-plant food items (fungi) and various plant structures (bark, root, seed) identified with microhistology could not be identified by DNA barcoding.

Both methods showed large variation in diets between individuals; the mean proportion of food items was often smaller than its standard deviation (Table 1 and 2).

## Discussion

Although there was an agreement between the methods with respect to the importance of the main plant groups, DNA barcoding gave by far a taxonomically more detailed picture of the diet of the two vole species than did the microhistological analysis. Indeed, much of the discrepancy in the results derived from the two methods could be explained by differences in taxonomic resolution. Other discrepancies must be attributed to particular biases in the methods. Because a substantial fraction of the stomach content is left unidentified by the microhistological diet analysis, the proportion of the different plant groups in the diets will be biased towards the most easily identified groups by this method. One example might be the fairly large discrepancy in the abundance of monocotyledons between the methods. Another bias is due to varying epiderm/mesophyll ratios between taxa. Finally, a general problem is introduced by a great deal of subjectivity in the microhistological identification processes so that experience of the observer will have a major impact on the outcome of the analysis.

However, quantitative comparison between the methods should be done cautiously. Quantitative interpretation of DNA barcoding results is not straightforward; partly because it is based on chloroplast DNA. Thus, species from which chloroplast-rich tissues are eaten, are likely to be overrepresented compared to species mostly represented by e.g. seed or root in the diet [31,46]. In addition, even if the *trn*L approach using *g-h *primers is universal for angiosperms and gymnosperms [33], the horsetails, mosses and fungi identified with microhistological method are mostly omitted by the DNA analysis. Together with easy microhistological identification this explains discrepancy between methods in the amount of *Equisetum*. Although DNA barcoding using the *trn*L approach also has its shortcomings, we conclude that it is superior to traditional methods for establishing diet analysis based on stomach content in small rodents. This novel method is less prone to the biases and context-dependencies that hamper microhistological analyses and yields a vastly improved taxonomic resolution. Information about diets at the level of individual plant species opens for testing more precise hypotheses on interactions between voles and plants. Furthermore, the new DNA-based technology makes it possible to study vole-plant interaction by non-destructive sampling of faeces in the natural habitats of voles. In faeces, DNA is highly degraded and only small fragments remain [47]. The short sequences obtained using the *trn*L approach (10–143 base pairs) allow applying it on faecal samples [31]. In this case, the first step will be to identify the rodent species using a mitochondrial DNA marker, and the second step will be the diet analysis. The analysis can even be more specific, by performing individual and sex identification using microsatellite polymorphism and Y-chromosome amplification [48,49]. Thus, diet comparisons among species, individuals and sexes can be carried out, even without observing the animals (e.g. [31]).

For increasing the resolution in genera where sequences do not vary among the species (e.g. *Carex *and *Salix*), the *trn*L approach using the *g-h *primers can be complemented by one or several additional systems, specially designed for amplifying a short and variable region in these genera (as suggested in Valentini et al. [31]). Another strategy would be to amplify the whole *trn*L intron using universal primers such as *c *and *d *designed by Taberlet et al. [40] (254–767 bp), which strongly increases the resolution [33]. However, even if this second strategy can be easily implemented for the analysis of stomach content (using the Titanium upgrade of the 454 FLX), it will not be suitable for faeces analysis because of the shortness of the degraded plant DNA fragments.

## Competing interests

The authors declare that they have no competing interests.

## Authors' contributions

EMS carried out the microhistological analysis, compared the results with DNA analysis and took the lead in writing the manuscript. AV, EC, CM and LG did the DNA analyses. CB, AKB and JHS provided the plant DNA database. RAI, NGY and PT developed the framework for the analyses. AV, LG, CB, AKB, RAI, NGY and PT assisted with the manuscript writing.
